# The PAICE suite reveals circadian posttranscriptional timing of noncoding RNAs and spliceosome components in *Mus musculus* macrophages

**DOI:** 10.1093/g3journal/jkac176

**Published:** 2022-07-25

**Authors:** Sharleen M Buel, Shayom Debopadhaya, Hannah De los Santos, Kaelyn M Edwards, Alexandra M David, Uyen H Dao, Kristin P Bennett, Jennifer M Hurley

**Affiliations:** Department of Biological Sciences, Rensselaer Polytechnic Institute, Troy, NY 12180, USA; Department of Biological Sciences, Rensselaer Polytechnic Institute, Troy, NY 12180, USA; The MITRE Corporation, Bedford, MA 01730, USA; Department of Biological Sciences, Rensselaer Polytechnic Institute, Troy, NY 12180, USA; Department of Mathematical Sciences, Rensselaer Polytechnic Institute, Troy, NY 12180, USA; Department of Biological Sciences, Rensselaer Polytechnic Institute, Troy, NY 12180, USA; Department of Mathematical Sciences, Rensselaer Polytechnic Institute, Troy, NY 12180, USA; Department of Computer Science, Rensselaer Polytechnic Institute, Troy, NY 12180, USA; Institute for Data Exploration and Applications, Rensselaer Polytechnic Institute, Troy, NY 12180, USA; Department of Biological Sciences, Rensselaer Polytechnic Institute, Troy, NY 12180, USA; Center for Biotechnology and Interdiciplinary Sciences, Rensselaer Polytechnic Institute, Troy, NY 12180, USA

**Keywords:** circadian rhythms, immunity, lncRNA, snRNA, omics

## Abstract

Circadian rhythms broadly regulate physiological functions by tuning oscillations in the levels of mRNAs and proteins to the 24-h day/night cycle. Globally assessing which mRNAs and proteins are timed by the clock necessitates accurate recognition of oscillations in RNA and protein data, particularly in large omics data sets. Tools that employ fixed-amplitude models have previously been used to positive effect. However, the recognition of amplitude change in circadian oscillations required a new generation of analytical software to enhance the identification of these oscillations. To address this gap, we created the Pipeline for Amplitude Integration of Circadian Exploration suite. Here, we demonstrate the Pipeline for Amplitude Integration of Circadian Exploration suite’s increased utility to detect circadian trends through the joint modeling of the *Mus musculus* macrophage transcriptome and proteome. Our enhanced detection confirmed extensive circadian posttranscriptional regulation in macrophages but highlighted that some of the reported discrepancy between mRNA and protein oscillations was due to noise in data. We further applied the Pipeline for Amplitude Integration of Circadian Exploration suite to investigate the circadian timing of noncoding RNAs, documenting extensive circadian timing of long noncoding RNAs and small nuclear RNAs, which control the recognition of mRNA in the spliceosome complex. By tracking oscillating spliceosome complex proteins using the PAICE suite, we noted that the clock broadly regulates the spliceosome, particularly the major spliceosome complex. As most of the above-noted rhythms had damped amplitude changes in their oscillations, this work highlights the importance of the PAICE suite in the thorough enumeration of oscillations in omics-scale datasets.

## Teaser

Post-transcriptional regulation plays an important role in timing circadian rhythms, yet the mechanisms of this regulation are poorly understood. To identify these mechanisms, we applied our novel Pipeline for Amplitude Integration of Circadian Exploration suite to jointly model rhythms in proteomics and transcriptomics data from *Mus musculus* macrophages. We revealed clock control of long non-coding RNAs, small nuclear RNAs, and the spliceosome complex, suggesting mRNA processing as an important post-transcriptional tuner of circadian rhythms.

## Introduction

Circadian rhythms are a cellular process that is observed across all taxa, ranging from cyanobacteria to humans (Kondo *et al.* 1993; Reddy *et al.* 2020). These near 24-h oscillations are coordinated by a molecular timekeeper, tightly conserved in metazoans and loosely conserved in bacteria and plants, that is entrained to a variety of external and internal cues, or zeitgebers. One of the strongest of these zeitgebers is the daily natural light and dark cycle, which synchronizes the transcription/translation negative feedback loop that comprises the circadian clock in eukaryotic organisms (Harmer *et al.* 2001). In mammals, circadian rhythms have been implicated in the control of sleep, digestion, immune function, and many other essential physiological mechanisms (Scheiermann *et al.* 2013; Morf and Schibler 2013; Chatterjee and Ma 2016; Valdez 2019; Segers and Depoortere 2021). The disruption of the circadian cycle has been linked to sleep disorders, obesity, cardiovascular illness, mental illness, stroke, and cancer, emphasizing the importance of the circadian clock in human health and well-being (Arble *et al.* 2009; Zhu and Zee 2012; Engin 2017; Thosar *et al.* 2018; Sulli *et al.* 2019; Stubblefield and Lechleiter 2019; Walker *et al.* 2020).

While the disruption of circadian rhythms is known to increase disease instances, the mechanisms by which this dysregulation drives negative health consequences are poorly understood. One hypothesis is that the disruption of circadian timing causes the dysregulation of the immune system, which in turn increases inflammation, thereby affecting many physiological processes (Comas *et al.* 2017; Collins *et al.* 2021). The most common molecular approach to infer disrupted circadian processes is to identify mRNA, protein, and other key elements of a cell that oscillate over circadian time using omics analysis (De los Santos *et al.* 2019, 2020, 2021). Analyzing these omics data to classify which elements in a cell are under the regulation of the circadian clock is a complex and ever-evolving task (Hughes *et al.* 2010, 2017). For example, though it was known that circadian oscillations often have a damping component, fixed-amplitude oscillatory models were standard for oscillatory identification, which are not designed to model these amplitude changes (ACs) (Robertson and Takahashi 1988; Guilding *et al.* 2009; Beer *et al.* 2017; Kawamoto *et al.* 2020). In addition, noisy data types, e.g. proteomics, hampered the identification of oscillations in omics data (Hurley *et al.* 2018; Collins *et al.* 2021).

In response to this issue, we created programs using models that can independently identify oscillations with ACs (Extended Circadian Harmonic Oscillator or ECHO), analyze the ontologies of each AC category (ECHO Native Circadian Ontological Rhythmicity Explorer or ENCORE), and use less noisy data to identify circadian oscillations in corresponding more noisy data (Multi-Omics Selection with Amplitude Independent Criteria or MOSAIC) (De los Santos *et al.* 2017, 2019, 2020, 2021). These individual programs have the capability to more accurately report the classification of genes and cellular pathways that undergo circadian regulation (De los Santos *et al.* 2020; Collins *et al.* 2021). They have also revealed the extent to which the amplitude of these oscillations changes over circadian time, which may play a role in circadian regulation (De los Santos *et al.* 2017).

As a part of this body of work, the effect of circadian timing on the mammalian macrophage was analyzed using transcriptome and proteome data gathered over the circadian day (Collins *et al.* 2021). While many mRNAs were found to be circadianly timed, little alignment between oscillatory mRNA and proteins in macrophages was noted, suggesting the presence of extensive posttranscriptional regulation in macrophages (Collins *et al.* 2021). This phenomena of circadian posttranscriptional regulation have been noted in many different organisms (Romanowski and Yanovsky 2015; Green 2018; Parnell *et al.* 2021). While there is strong evidence of circadian posttranscriptional regulation, there is a limited understanding of what molecular mechanism(s) coordinate this regulation. Relevantly, noncoding RNA (ncRNA) and the spliceosome are known to be indispensable regulators of protein expression and cell processes and could play a role in circadian posttranscriptional regulation (Will and Lührmann 2011). Previous work recognized that these ncRNAs may play regulatory roles in circadian output (Cui *et al.* 2015; Fan *et al.* 2017; Hardeland 2020). However, a global analysis exploring the effect of circadian transcriptional regulation on total ncRNA levels, and how this could affect circadian posttranscriptional regulation, has not been completed.

To close this critical gap in the field, we combined our ECHO, ENCORE, and MOSAIC programs into the Pipeline for Amplitude Integration of Circadian Exploration (PAICE) suite and applied the power of the PAICE suite to our macrophage omics data with a particular focus on the ncRNAs. To aid future use in the field, we provided a comprehensive tutorial of the PAICE suite and its application to circadian omics data. Exploring our previously published macrophage data with the PAICE suite, we noted novel relationships between circadianly regulated transcripts and proteins in murine macrophages (De los Santos *et al.* 2020; Collins *et al.* 2021). We found that noncoding genes are highly regulated by the circadian clock in macrophages, many of which play roles in metabolism or are implicated in human disease. We also identified that the small nuclear RNA (snRNA), and constituent coding transcripts and proteins of the major spliceosome complex, undergo extensive circadian regulation, suggesting that the spliceosome may be a source of circadian posttranscriptional regulation in macrophages.

## Materials and methods

### ECHO analysis of transcripts and proteins

We used the PAICE suite to extend the results of Collins *et al.* (2021). Briefly, Collins *et al.* (2021) investigated rhythmic patterns in the transcriptome and proteome of the mouse macrophage. Bone marrow-derived monocytes harvested from male PER2::LUC mice (C57BL/6J) were differentiated into macrophages, grown to confluence, and then subjected to a 2-h serum shock to synchronize their circadian rhythms. Samples were collected every 2 h over a 48-h period with 3 replicates at each time point and analyzed using RNA-seq and TMT-MS (Collins *et al.* 2021). All hit counts from RNA-seq data were normalized to transcripts per million (TPM). LIMBR (Learning and Imputation for Mass-spec Bias Reduction) was used to remove any transcripts or proteins that were detected in less than 70% of the samples, impute missing values, and adjust for batch effects (Crowell *et al.* 2019). Transcripts and proteins were free-run (i.e. no period restrictions) through ECHO v3.0 to determine rhythmicity with additional adjustments for *z*-score normalization, linear de-trending, and data smoothing. Deviating from Collins *et al.*’s (2021) use of ECHO v3.0, we used ECHO v4.0 to determine circadian genes. These results were restricted postrun to period parameters of 20–28 and 18–30 h for transcripts and proteins respectively, and selected for significance based on a BH-adjusted *P*-value cutoff of <0.05. The resulting files were used as the input for all analyses of transcripts and proteins in this manuscript with the exception of MOSAIC analysis, where LIMBR-adjusted data were directly used (see below) (Collins *et al.* 2021) (Supplementary Files 1 and 2). To maintain consistency across gene naming conventions, 123 proteins were manually remapped or deleted preceding further analysis as labeled in Supplementary File 3.

### ENCORE analysis of transcripts and proteins

The ECHO results were analyzed through ENCORE v4.0 using the ECHO period parameters for transcripts and proteins respectively (Supplementary Files 4 and 5). This exploration through the ENCORE tool allowed for significantly more sophisticated ontological analysis than that in Collins *et al.* (2021), due to ENCORE’s integration of Gene Ontology Enrichment, STRING, QuickGO, UNIPROT, and advanced visualizations (Ashburner *et al.* 2000; Binns *et al.* 2009; Szklarczyk *et al.* 2017; UniProt Consortium 2019; Collins *et al.* 2021). Consistent with the ECHO analysis, a BH-adjusted *P*-value cutoff of <0.05 was used to determine significance. A BH adjustment was used in place of the Bonferroni correction often deployed in the PANTHER tool, as the Bonferroni correction in PANTHER is adjusted from the traditional multiplicative adjustment to account for tree dependency, which is not the implementation available in R (Thomas *et al.* 2003). As such, we mitigated this by using a less strict adjustment.

### MOSAIC analysis of transcripts and proteins

LIMBR-adjusted, but not ECHO-analyzed, transcript and protein data were analyzed in MOSAIC v0.2.4 to jointly model the transcriptome and proteome (Supplementary File 6). To maintain consistency with ECHO/ENCORE analysis, MOSAIC joint analysis was free-run and then restricted postrun. A BH-adjusted *P*-value cutoff of >0.05 was entered as the statistical significance threshold in MOSAIC. Each component of the PAICE suite and how they were used to enhance previous work is further described in the PAICE Suite Tutorial.

### lncRNA disease analysis

In addition to the analysis of the coding portions of the transcriptome and proteome, we analyzed the noncoding transcriptome. Raw TPM values for all analyzed ncRNA are available (Supplementary File 7). The LIMBR-adjusted data for all noncoding transcripts were free-run through ECHO with the parameters described above to find rhythmic ncRNA. This gene set was then further narrowed to a period of 20–28 h and a BH-adjusted *P*-value of <0.05. These resulting significant circadian ncRNA transcripts were sub-classified into AC categories, and a Fisher’s exact test was used to determine whether each AC category was enriched for mRNA or ncRNA. Further analysis stratified the ncRNA into more detailed subtypes [long noncoding RNA (lncRNA), pseudogene, small nucleolar RNA (snoRNA), snRNA, etc.], and the Fisher’s exact test was repeated to determine whether each subtype was enriched for circadian or noncircadian gene expression. We next compared the list of circadian lncRNA with the lncRNA disease database LncRNADisease v2.0 to find which lncRNAs are both circadian and have an experimentally determined role in disease (Bao *et al.* 2019) (Supplementary Files 8–10).

### snRNA and spliceosome analysis

Information from the Kyoto Encyclopedia of Genes and Genomes (KEGG) pathways database and ECHO was used to manually identify transcripts in the spliceosomal pathway that were circadianly regulated (Kanehisa *et al.* 2016; De los Santos *et al.* 2020) (Supplementary Files 11 and 12). SnRNAs were manually identified from the NCBI Nucleotide database and classified into their respective spliceosomal category (Supplementary File 13). All rhythmic snRNA were further segregated into their respective AC categories as identified by ECHO. All transcripts, proteins, and snRNA were graphed using Prism 9.1.1 (GraphPad Software, San Diego, California USA, www.graphpad.com), and figures were created with and assembled in BioRender.com.

## Results

### Tutorial for the application of the PAICE suite to circadian omics data

**Fig. 1.**
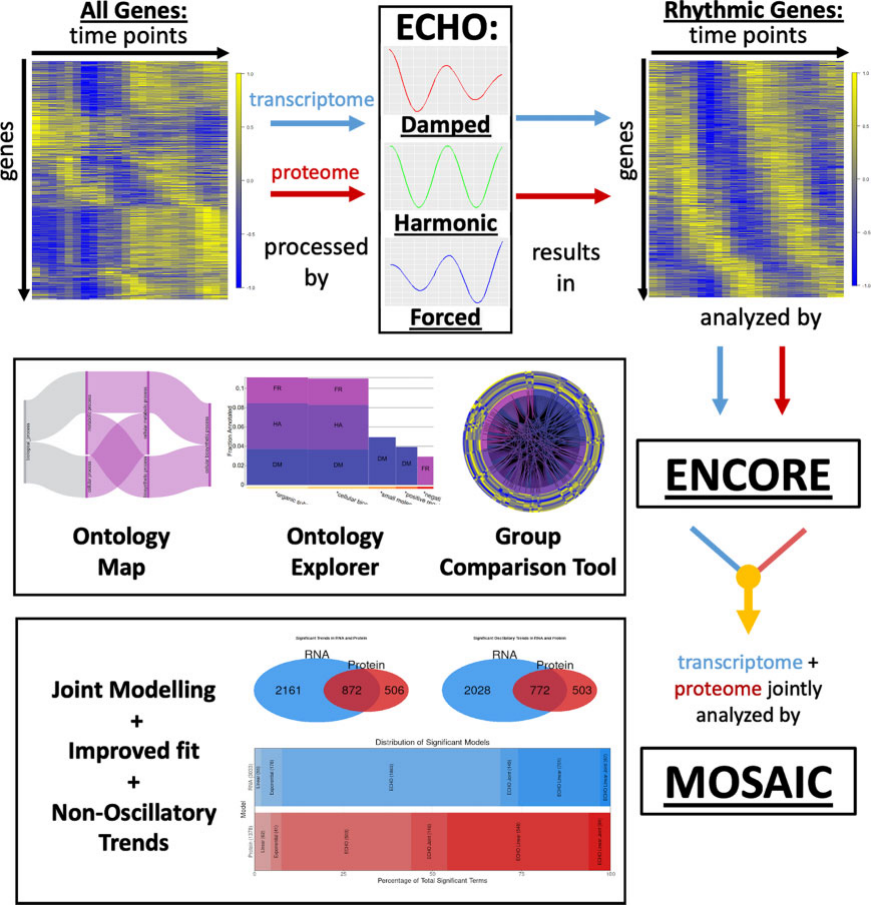
The work flow of the PAICE suite. After ECHO detects oscillations in time-resolved omics data, ENCORE combines information from GO, STRING, QuickGO, and UniProt to create visualizations related to the functional categories of the ECHO-identified oscillating elements, taking the AC into account. MOSAIC improves upon the ECHO fit, identifies nonoscillatory trends, and jointly models transcriptomic and proteomic data from the same sample to dampen the effect of biological or analytical noise. Representative data shown analyzed by the PAICE suite from Collins *et al.* (2021), Li *et al.* (2019), and this work.

To combine the individual powers of ECHO, ENCORE, and MOSAIC, we created the PAICE suite (Fig. 1). The PAICE suite is designed to identify, visualize, analyze, and contextualize circadian rhythms in a high-throughput fashion in the context of large omics data sets (De los Santos *et al.* 2019, 2020, 2021). This suite of R programs, freely available on GitHub (https://github.com/delosh653/ECHO, https://github.com/delosh653/ENCORE, https://github.com/delosh653/MOSAIC) and as R packages (https://cran.r-project.org/web/packages/echo.find/vignettes/echo-vignette.html, https://cran.r-project.org/web/packages/mosaic.find/vignettes/mosaic-vignette.html), is operated via web-browser-based shiny apps that offer a variety of point-and-click options to assist in the ease of use, allowing users to customize the data analysis options to best suit their data and interests.

The first program of the data analysis pipeline, ECHO, identifies and categorizes statistically significant oscillations into 3 AC categories: damped, harmonic, and forced (Fig. 1). To begin analysis, omics data are entered into ECHO’s “Finding Rhythms” tab where users may then select appropriate data parameters (such as time course length and resolution and number and type of replicates), various statistical corrections, preprocessing categories, and the temporal period of interest. The ECHO results are summarized in the ECHO output file, which displays the AC, period, and phase shift of each individual gene, among other descriptors. Users may download and input this file into the “Visualizing Results” tab to generate heat maps, gene lists, expression graphs, and Venn diagrams of the discovered oscillations.

The second step of the analysis pipeline, ENCORE, creates ontological visualizations from the ECHO results. The ECHO output file generated from the initial analysis must be entered into ENCORE’s “Create ENCORE File” tab to use the app. To leverage ENCORE’s integration of Gene Ontology (GO) Enrichment, STRING, QuickGO, and UNIPROT databases, users must select an organism name, gene ID type, and other parameters that describe the data (Ashburner *et al.* 2000; Binns *et al.* 2009; Szklarczyk *et al.* 2017; UniProt Consortium 2019). These user-entered specifications will produce an ENCORE file to be downloaded and inputted into the “Explore” tab. The Explore Tab hosts the 3 core ontological visualizations: the “Ontology Map,” the “Ontology Explorer,” and the “Group Comparison Tool” (Fig. 1). The Ontology Map is an interactive Sankey diagram with ontological pathways colored by AC category. Users may click on any GO term in the Ontology Map to load the Ontology Explorer and Group Comparison Tool with data about the selected term’s ontological children. The Ontology Explorer is an interactive histogram that shows the fold enrichment, AC breakdown, and fraction annotated for each displayed GO term. Users may click on the node under any GO term to advance the Ontology Explorer to the next hierarchy of ontological children. The Group Comparison Tool is an interactive chord diagram where a circular heatmap of ontologically related gene expression appears along the outer edge. The inner chords visualize STRING protein–protein interactions, allowing the user to explore how circadian oscillation affects the connected functionality. Users may also access an “Auxiliary Information” tab to explore expression graphs, ECHO fit, and phase-shift for all genes involved in the selected GO term.

While the ECHO and ENCORE applications can analyze individual types of omics data, the MOSAIC application jointly models less and more noisy data that are correlated (e.g. transcriptomic/proteomic data or proteomic/metabolomic data) to improve oscillatory model fit, visualize continuity between corresponding data (e.g. a coding transcript and its protein), and identify nonoscillatory trends. MOSAIC also has an expanded library of models to describe a greater breadth of oscillation than the ECHO application. Linear and exponential trends indicate that a nonoscillatory trend is detected, signifying a pattern of gene expression that is distinct from circadian regulation, and MOSAIC fits the basic mathematical definition of these line types. The ECHO trend indicates that one of the 3 types of AC oscillation is detected (damped, harmonic, or forced). If oscillation in a gene is detected, but MOSAIC’s joint modeling is needed to detect it, it is classified as ECHO Joint. The ECHO Linear trend models genes that exhibit both oscillation and a linear trend, with oscillations that increase in a linear manner, possibly due to coregulation of noncircadian and circadian mechanisms. Finally, ECHO Linear Joint models demonstrate the above-described ECHO Linear trend but can only be detected by the joint modeling of the 2 related omic data sets with MOSAIC (De los Santos *et al.* 2021). To use MOSAIC, the LIMBR-adjusted omics data from the more and less noisy data types must be inputted through the MOSAIC “Finding Rhythms” tab. As with ECHO, the user is able to describe the data parameters and select their preferred statistical tests. The resultant MOSAIC output file can be uploaded into the “Visualizing Results” tab. This tab provides the user with joint expression graphs, AC coefficient density plots, and heatmaps. In addition, MOSAIC visualizes a distribution of significant trends, allowing the user to explore the relationships between oscillatory and nonoscillatory genes. Uniquely, these features are able to display similarities and differences in expression patterns between corresponding data.

### Joint modeling of the transcriptome and proteome with the PAICE suite improves the identification of circadian multiomic oscillations

**Fig. 2.**
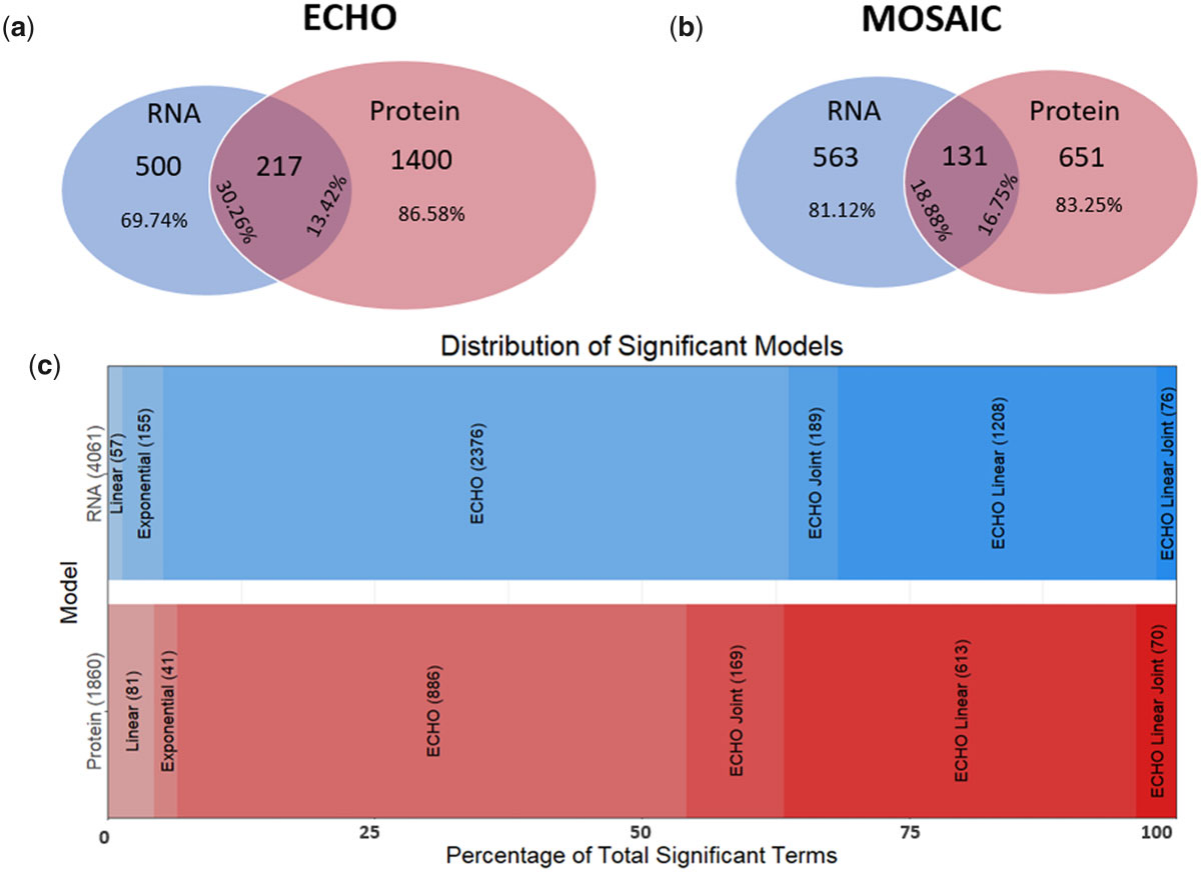
MOSAIC analysis suggests posttranscriptional regulation in macrophages. a) Venn diagram comparing statistically significant (BH-adj *P*-value <0.05) circadian RNA (period = 20–28) and proteins (period = 18–30) identified by ECHO in the transcriptome and proteome. LIMBR-adjusted data were filtered for genes that have detectable mRNA and protein expression. Relative percentages of circadian RNA that has no corresponding protein, circadian RNA with corresponding protein, circadian protein with corresponding RNA, and circadian protein that has no corresponding RNA are listed (L–R). b) Venn diagram comparing statistically significant (BH-adj *P*-value <0.05) circadian RNA (period = 20–28) and proteins (period = 18–30) identified by MOSAIC in the transcriptome and proteome. MOSAIC automatically filtered LIMBR-adjusted data for genes with detectable expression at both mRNA and protein levels. Relative percentages of circadian RNA that has no corresponding protein, circadian RNA with corresponding protein, circadian protein with corresponding RNA, and circadian protein that has no corresponding RNA are listed (L–R). c) Bar graph comparing the distribution of significant trends in the transcriptome and proteome as defined by MOSAIC. Each bar graph is divided into segments that represent linear, exponential, ECHO, ECHO Joint, ECHO Linear, and ECHO Linear Joint trends, respectively, and each segment is labeled with the number of genes in the category (De los Santos *et al.* 2021). The width of each category represents the percentage of each category in comparison to the total number.

To investigate circadian posttranscriptional regulation, Collins *et al.* (2021) used ECHO to identify circadian oscillations in the murine macrophage transcriptome and proteome, reporting significant discrepancies (Collins *et al.* 2021). However, due to technical limitations, higher rates of technical noise from the proteomic data sets could have introduced false negatives into this comparison (Hurley *et al.* 2018). We therefore analyzed the data from Collins *et al.* (2021) using MOSAIC to determine if the wide discrepancy between oscillations in the transcriptome and proteome was maintained when the data were jointly modeled (Fig. 2) (Supplementary File 6). For reference, we provide the gene *Eifb23* as an example of a possible false negative in ECHO analysis. Independent ECHO analysis of the *Eifb23* transcript revealed an oscillation (*P*-value = 1.48 × 10^9^), but the protein did not (*P*-value = 0.93). MOSAIC revealed that the root mean squared error, an estimate of noise, was 0.24 for the transcript and 0.35 for the protein and identified an oscillation in EIFb23 protein (*P*-value = 2.75 × 10^8^). In addition, as MOSAIC automatically omits any terms that do not have detectable expression at both omic levels (i.e. a gene must have both detectable mRNA and protein expression to be analyzed), it was necessary to manually filter the LIMBR-adjusted data in the same manner before entering the data into ECHO for proper comparison.

For both the ECHO and MOSAIC analyses, 20–28 and 18–30-h period restrictions were applied to determine circadian trends in the transcriptome and proteome respectively. CT was calculated based on peak and trough *Per2* expression of ex vivo peritoneal macrophages as reported in Keller *et al.* (2009). Our ECHO analysis closely resembled the data from Collins *et al.* (2021) (Fig. 2a). By comparison, MOSAIC found fewer total circadian terms (Fig. 2b), but a greater percentage of circadian proteins with circadian transcripts (Fig. 2, a–c). This suggests both that noise from the proteomic data could introduce false positive and false negative oscillatory trends and that, as predicted, MOSAIC is able to use correlated data with less technical noise to infer oscillations more accurately.

However, even with an increase in the relative overlap of proteins, there were still many proteins that oscillated without an oscillating transcript, suggesting that posttranscriptional regulation is an important part of circadian timing in macrophages (Fig. 2b). Furthermore, while in general the phases of oscillating transcripts were more aligned with the phases of the oscillating proteins in the MOSAIC analysis (Supplementary Fig. 1, a and c), phase delays noted in the Collins *et al.* (2021) data set were maintained whether ECHO or MOSAIC was used to analyze the data (Supplementary Fig. 1, b and d).

### The PAICE suite enhances ontological analysis of the murine coding transcriptome and proteome

**Fig. 3.**
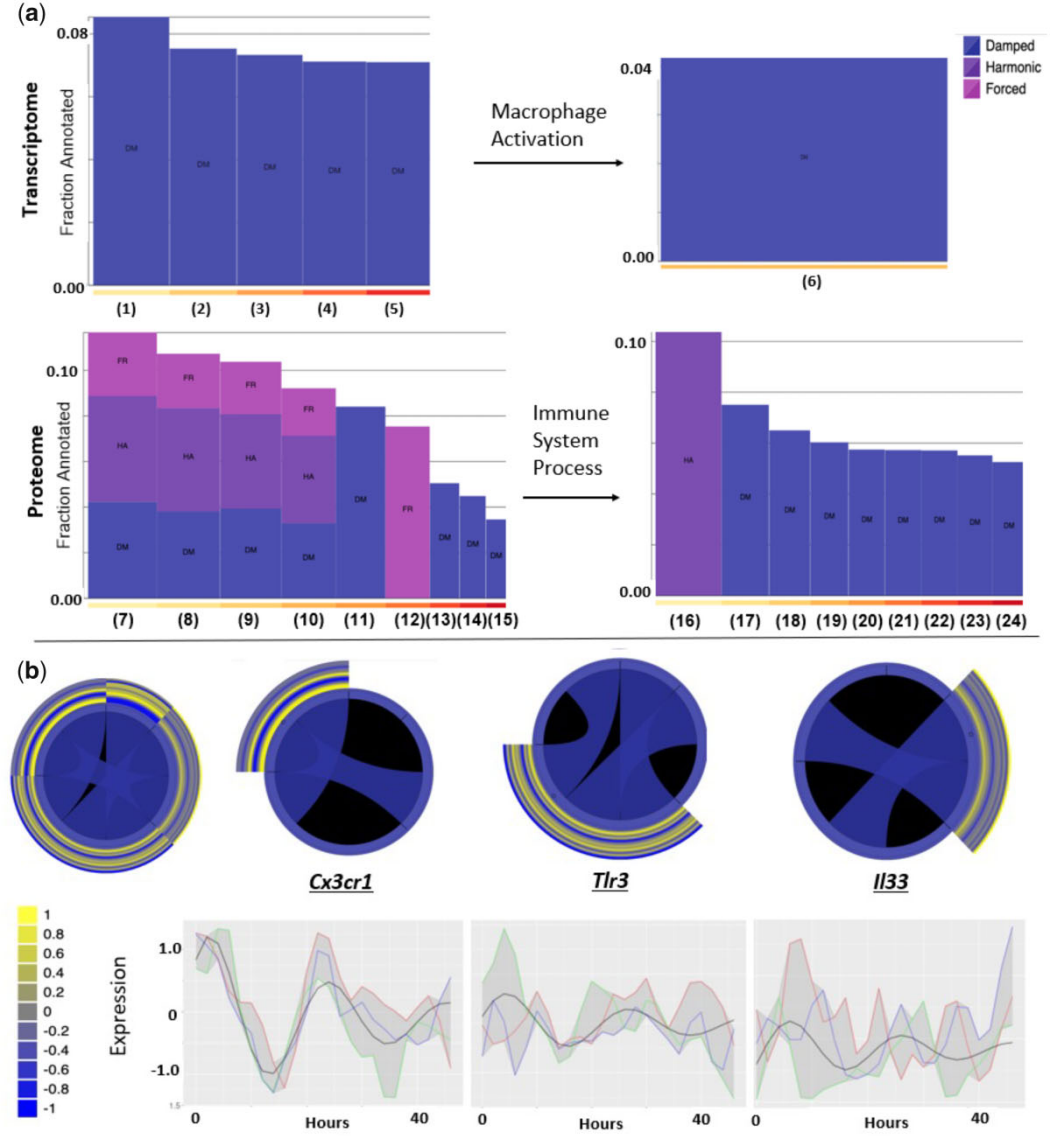
ENCORE highlights differences in both ontologies and AC categories between the macrophage transcriptome and proteome. a) Bar plots displaying the AC categories of enriched ontologies in the macrophage transcriptome and proteome (DM, damped; FA, forced; HA, harmonic). The fraction annotated of each ontological term is plotted on the *y*-axis, with ontological terms abbreviated as follows: (1) locomotion, (2) developmental process, (3) metabolic process, (4) biological regulation, (5) cellular process, (6) macrophage activation involved in immune response, (7) cellular component organization, (8) metabolic process, (9) localization, (10) cellular process, (11) cell killing, (12) pigmentation, (13) immune system process, (14) biological adhesion, (15) developmental process, (16) antigen processing and presentation, (17) myeloid cell homeostasis, (18) immune effector process, (19) positive regulation of immune response, (20) regulation of immune system process, (21) leukocyte activation, (22) negative regulation of immune response, (23) immune response, and (24) immune system development. b) ENCORE’s Group Comparison Tool reveals chord diagrams of mRNAs involved in the macrophage activation ontology and the related expression graphs. An overall and sectioned (by gene) chord diagram for oscillating genes in the macrophage activation gene ontology. The first chord diagram is the overall chord diagram and gene connections, and each subsequent diagram represents one of the 3 specific genes and its connections in the macrophage activation gene ontology, *cx3cr1*, *tlr3*, and *il33*. The heatmaps surrounding the chord diagram represent the oscillatory nature of each of the genes. The relative expression of each of the transcripts, as analyzed by ECHO, is displayed below each of the chord diagrams.

Collins *et al.* (2021) noted that 15.3% of the macrophage coding transcriptome was modeled by an approximately 24-h oscillation using ECHO and that these oscillations were biased toward a damped waveform, rather than harmonic or forced waveforms (Collins *et al.* 2021). We hypothesized that the damping of these rhythms may have a coordinated effect on the regulation of circadian output. Therefore, to understand the effect of the damping of circadian rhythms on macrophage physiological processes, we applied the PAICE suite, particularly ENCORE’s visualizations, to the Collins *et al.* (2021) data (Supplementary File 4). ENCORE predicted that categories enriched in oscillatory genes were exclusively modeled by damped waveforms and discovered several enriched ontologies that were not found in Collins *et al.* (2021) (Fig. 3a).

As Collins *et al.* (2021) showed how the circadian production of ATP regulated the immune response ex vivo (Collins *et al.* 2021), we explored this process further using ENCORE to focus on transcripts within immunometabolic ontologies that undergo circadian oscillations. We found few enriched ontologies under the general immune system processes ontology. However, the downstream ontology term, macrophage activation, was enriched for genes under circadian regulation at the level of the transcriptome. The ENCORE Gene/Term Explorer revealed that the 3 circadianly regulated transcripts in this ontology, *cx3cr1*, *tlr3*, and *il33*, were highly connected genes in this term (Fig. 3b). Using the chord diagram feature of ENCORE, we showed how these 3 genes interacted with one another and the clock coordinated the phase of their oscillation so that the peak phases occurred at the same time during the circadian cycle, suggesting that this pathway is targeted for tight circadian regulation.

Collins *et al.* (2021) further described that 29% of the proteome was circadianly regulated, noting that damping was more widespread in the proteome than in the transcriptome (Collins *et al.* 2021). Using ENCORE, we found that while there was widespread damping in the proteome, unlike in the transcriptome, many of the highly enriched processes contained proteins from all 3 AC categories (Fig. 3a) (Supplementary File 5). Relevantly, genes in the ontological category that included immune system processes were predominantly damped. Further, investigation of this categories’ ontological subcategories, including myeloid cell homeostasis, immune effector processes, positive regulation of immune system processes, and leukocyte activation, showed that these subcategories were also damped (Fig. 3a). Relevantly, the immune system processes ontology was predominantly damped in its ontological subprocesses, including myeloid cell homeostasis, immune effector processes, positive regulation of immune system processes, and leukocyte activation (Fig. 3a). These data suggest that immune responses are broadly damped in these culture conditions, linking a decrease in available bioproducts (potentially energy) to a reduction in oscillations in the immune response, as was suggested by Collins *et al.* (2021).

### Circadianly regulated lncRNAs are implicated in human disease

While the murine macrophage data set from Collins *et al.* (2021) contained information on ncRNAs, the effect of circadian regulation on these ncRNAs was not reported (Collins *et al.* 2021). This omission is important as there are little data on the circadian regulation of ncRNAs, though what is known points to a clock effect on ncRNAs and their posttranscriptional regulation (Cui *et al.* 2015; Fan *et al.* 2017; Hardeland 2020). Therefore, to investigate clock effects on ncRNAs, we analyzed the transcriptome from Collins *et al.* (2021) using the PAICE suite to identify ncRNAs oscillating with a circadian period in murine macrophages (Collins *et al.* 2021) (Supplementary Files 8–12). The filter referenced in Fig. 2a was expanded to include the entire LIMBR-adjusted data for ECHO analysis in this section, including the ncRNAs.

**Fig. 4.**
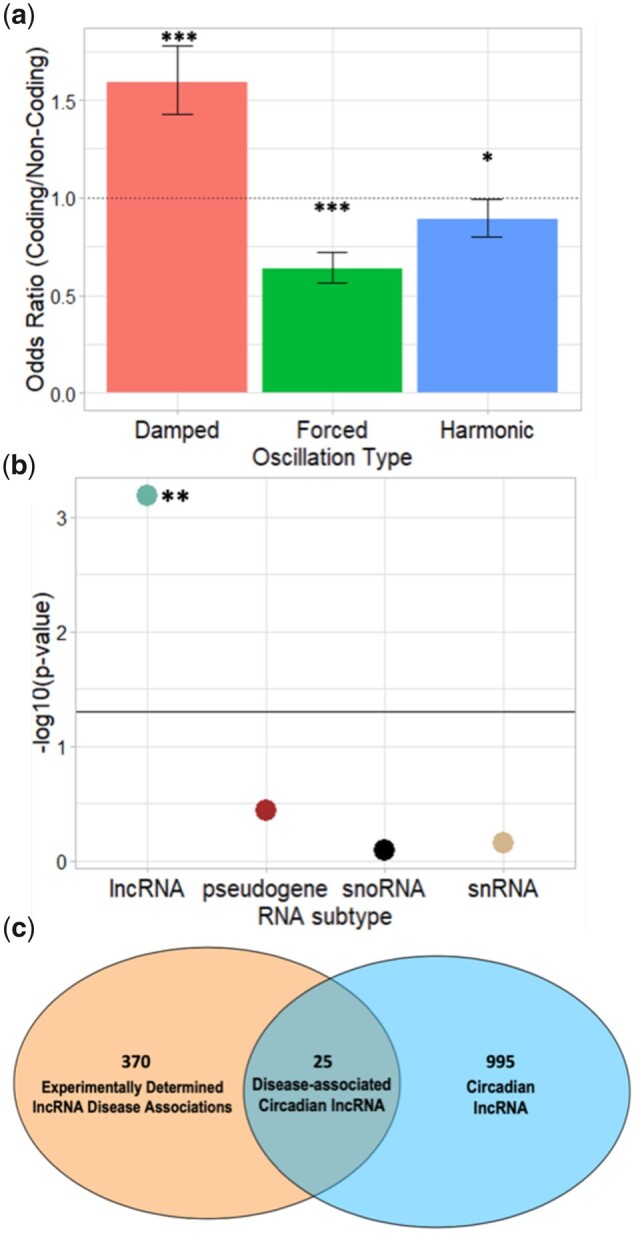
The PAICE suite identifies disease-linked ncRNAs under circadian regulation. a) Bar plot comparing the likelihood of finding coding genes over noncoding genes in each AC category (Fisher’s exact test odds ratio). b) Manhattan plot of the likelihood of identifying circadian oscillations in each ncRNA category. Statistically significant categories exceed the threshold of *P* = 0.05 (Fisher’s exact test). c) Venn diagram displaying the overlap of circadian lncRNAs identified in murine macrophages and disease-associated lncRNAs from LncRNADisease v2.0. In (a) and (b), statistical significance is indicated as follows: *P* < 0.05*, *P* < 0.001**, *P* < 2.2e−16***.

In total, we identified 2,765 out of 16,236 ncRNAs (17.03%) oscillated with a circadian period. Compared to oscillating mRNA, ncRNAs were more likely to have a forced or harmonic oscillation (Fisher’s exact test, damped oscillation odds ratio = 1.59, *P*-value <2.2 × 10^−16^, forced oscillation odds ratio = 0.64, *P*-value = 3.86 × 10^−13^, harmonic oscillation odds ratio = 0.89, *P*-value = 0.04) (Fig. 4a). We next stratified the ncRNAs into long lncRNA (which includes antisense lncRNA, sense lncRNA, bidirectional promoter lncRNA, long intergenic lncRNA, sense intronic lncRNA, and sense overlapping lncRNA), pseudogene RNA, snoRNA, and snRNA subgroups. Smaller ncRNAs, such as miRNA, were not included in this analysis given the sequence length limitations of the RNAseq approach used. We found that lncRNA was the only ncRNA enriched in circadian transcripts over noncircadian transcripts (Fisher’s exact test, odds ratio = 1.14, *P*-value = 6.45 × 10^−4^) (Fig. 4b). Out of 5,691 lncRNA transcripts, ECHO identified 995 (17%) of those as having significant circadian oscillations (Supplementary Fig. 2). Given the enrichment for oscillation among lncRNAs and their role in human disease (particularly inflammatory diseases), we next explored the role of circadian regulation of disease-associated lncRNAs by comparing the 995 oscillating lncRNAs to the lncRNA disease database LncRNADisease v2.0 (Bao *et al.* 2019; Chen *et al.* 2019; Zhang *et al.* 2019). Out of the 370 experimentally determined disease-associated lncRNAs, 25 (16 unique) disease-associated lncRNAs were found to oscillate with a circadian period (Fig. 4c) (Supplementary File 9). These lncRNAs play roles in the epigenetic regulation of anxiety, Parkinson’s disease-related inflammation, cancer, cardiac and liver disease, septicemia, and neurodegenerative disease (Poffenberger *et al.* 2010; Spadaro *et al.* 2015; Huang *et al.* 2017; Sunwoo *et al.* 2017; An *et al.* 2018; Cao *et al.* 2018; Zhu *et al.* 2018; Shin *et al.* 2019; Bu *et al.* 2020). A query for human orthologs of these oscillating lncRNAs was also conducted, revealing 4 additional disease relevant lncRNAs for future exploration (Supplementary File 10).

### Key regulatory components of the spliceosome pathway are circadianly regulated

While lncRNAs were the only ncRNA subclass enriched over background, we found several categories of ncRNAs that were regulated extensively by the circadian clock. One such class was snRNAs, which are essential for the proper recognition of mRNA-binding sites for splicing. snRNAs bind with the protein complexes of the spliceosome pathway to form small nuclear ribonucleoproteins (snRNPs) to guide the complex to bind exon-intron sites on pre-mRNA (Morais *et al.* 2021). snRNAs have also been previously suggested to be under circadian control in several model organisms (Perez-Santángelo *et al.* 2014; Schlaen *et al.* 2015; Aitken and Semple 2017; Ma *et al.* 2019).

**Fig. 5.**
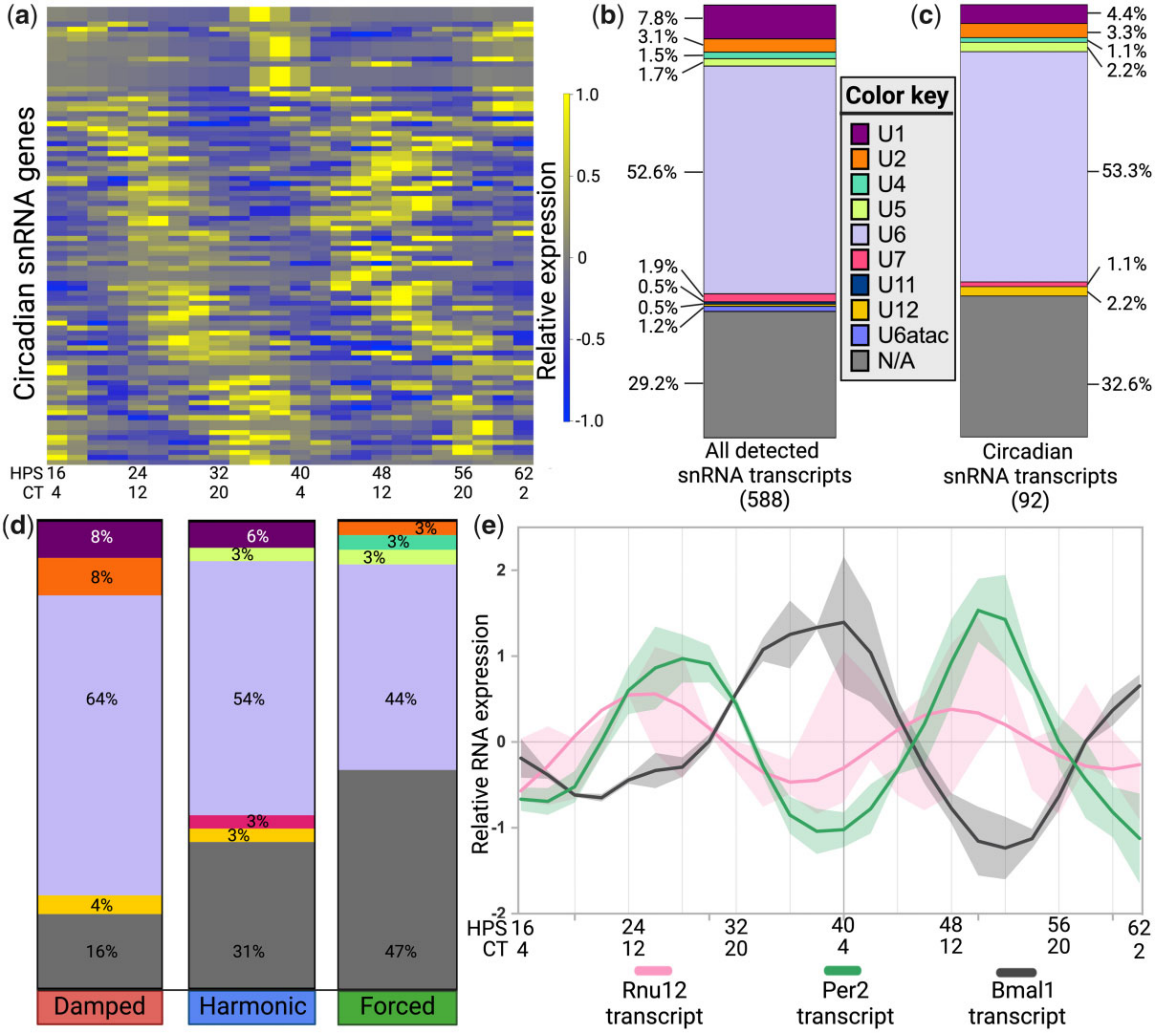
The circadian clock regulates snRNA expression. a) Heatmap displaying the relative (*Z*-scored) expression of circadian snRNAs as identified by the PAICE suite (period = 20–28 h, BH-adjusted *P*-value <0.05). HPS, hours following serum shock for circadian synchronization as in Collins *et al.* (2021); CT, circadian time based on peak and trough *Per2* expression of ex vivo peritoneal macrophages as reported in Keller *et al.* (2009). b) Bar graph displaying the % distribution of snRNA categories in all detected snRNA transcripts, with gene numbers indicated in parentheses below the bar graph legends. c) Bar graph displaying the % distribution of snRNA categories in all oscillating snRNA transcripts. d) Distribution of oscillating snRNA transcripts across 3 AC categories by snRNA category (*n* = 92 total genes, damped *n* = 25 genes, harmonic *n* = 35 genes, forced *n* = 32 genes). e) Expression of ECHO-fitted Rnu12 transcript (period = 23.1 h, BH-adj *P*-value = 4.18E−4), LIMBR-adjusted *Per2* transcript (period = 23.1 h, BH-adj *P*-value = 3.89E−18), and *Bmal1* transcript (period = 29.3 h, BH-adj *P*-value = 3.96E−20). Dark lines represent the ECHO model while shading represents the standard deviation of the data. The color key references snRNA categories in (b)–(d).

From our PAICE suite analysis, 92/588 (15.6%) snRNA transcripts were identified to oscillate with a circadian period (Fig. 5a) (Supplementary File 12). We next used NCBI Nucleotide to manually categorize all the snRNA transcripts into the well-studied classes of snRNA: U1, U2, U4, U5, and U6 (associated with the major spliceosome), U11, U12, U4atac, and U6atac (associated with the minor spliceosome), and U7 (histone pre-mRNA processing) (Fig. 5, b and c) (Supplementary File 13) (Morais *et al.* 2021). The stratified circadian snRNAs were then categorized by AC category (damped, harmonic, or forced) (Fig. 5d). Two classes, U11 and U6atac, which had low numbers in each class, were not represented in the circadian transcripts. Of the remaining classes, we found no enrichment of circadian oscillations (Fig. 5, b and c). The largest class, U6, displayed all 3 waveforms, but most U6 snRNAs were damped. U1 and U12 snRNAs only oscillated with damped or harmonic waveforms, and the U2 snRNAs were either damped or forced (Fig. 5d). *Rnu12*, the only circadian snRNA to have been studied in depth and a member of the U12 snRNA family, has a damped waveform and oscillates antiphase to Bmal1 transcript and in-phase with *Per2* transcript, suggesting direct regulation of *Rnu12* by BMAL1 (Fig. 5e). Notably, mutations of *Rnu12* detected in monocytes lead to early onset of cerebellar ataxia, a neurodegenerative disease that is known to correlate with sleep disruption (Velázquez-Pérez *et al.* 2011; Elsaid *et al.* 2017; Werdann and Zhang 2020).

While the major snRNA classes were broadly represented in the forced category, no minor snRNAs displayed forced waveforms. Splicing is a highly ATP-dependent process, with ATP required for nearly every step (Shi 2017). During nutrient-limiting conditions, macrophages adjust their transcriptional programs to adapt to their environment and the damping of the minor spliceosome may be part of that process. Limiting oscillations in the minor spliceosome components may be a way for macrophages to sequester ATP for the essential energy-demanding splicing reactions by the major spliceosome as the media is depleted in our culture conditions. In an exception to this, however, U7 snRNAs displayed consistently harmonic circadian oscillations (Fig. 5c). As the primary function of U7 snRNAs is to bind histone pre-mRNA within the U7 snRNP in order for splicing to occur, this suggests that the recognition of histone transcripts for splicing is resistant to the depletion of the media in our culture conditions (Dominski and Marzluff 2007).

**Fig. 6.**
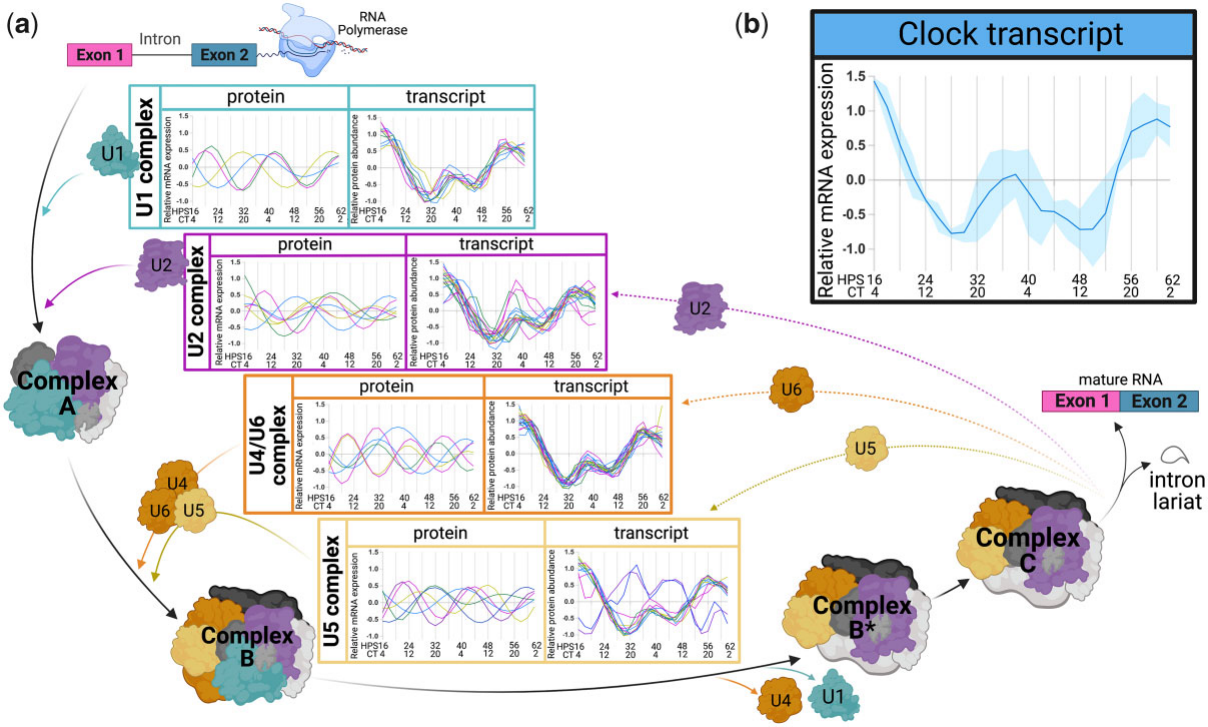
Circadian oscillations in the transcripts of the components of the spliceosome pathway mirror that of the *clock* transcript. a) Schematic representation of the canonical spliceosome pathway with the transcripts and proteins in each major complex aligned with the first step in which the complex plays a role in splicing. A newly transcribed pre-RNA binds to members of the U1 and U2 complexes to create complex A. The preassembled U4/U6 and U5 tri-snRNP complexes bind to complex A to create complex B, which, after dissociating from the U1 and U4 complexes, forms activated complex B*. Complex B* and subsequent complex C actively cleave and excise the intron lariat, thereby joining exons 1 and 2 to create the mature RNA, releasing the U2, U5, and U6 complexes, which are recycled back into the spliceosome pathway. The graphs for each major complex (U1, U2, U4/U6, U5) highlight the transcripts and proteins within each respective complex that oscillate with a circadian period, with transcript plots using post-LIMBR data points and protein plots showing ECHO-fitted curves. b) The expression of the *clock* transcript from the Collins *et al.* (2021) macrophage data set. HPS, hours following serum shock for circadian synchronization as in Collins *et al.* (2021); CT, circadian time based on peak and trough Per2 expression of ex vivo peritoneal macrophages as reported in Keller *et al.* (2009).

Corresponding to our identification of circadian oscillations among snRNAs, Collins *et al.* (2021) noted circadian oscillations in many members of the spliceosome complex at the protein level, with a peak in the late circadian day (CT15–CT3) (Collins *et al.* 2021). As the spliceosome pathway could play a role in the posttranscriptional regulation of circadian output, we next investigated oscillations of both the proteins and their corresponding mRNAs which are involved in the spliceosome complex using the PAICE suite (Fig. 6) (Supplementary File 13). We identified 57/64 (89.06%) of transcripts that code for proteins in the major spliceosome complexes U1, U2, U4/U6, and U5 oscillated with a circadian period, though a *P*-value could not be assigned due to the unusual AC patterning of these genes. Notably, the characteristic waveform for these spliceosomal mRNAs closely aligned with the cycling patterning of the transcript of *clock*, one of the main regulatory genes of the positive arm of the circadian clock, suggesting a direct relationship between the expression of *clock* and the expression of the members of the spliceosome complex (Fig. 6b).

All transcripts oscillated in-phase with each other save 2, the U5 transcripts *Prpf6* and *Snrnp200* (Fig. 6a). *Prpf6* is essential in creating binding stability among the proteins of the tri-snRNP complex (U4/U6.U5) in order for complex B to form and, consequently, for splicing to proceed, suggesting that the clock may use *Prpf6* to time the formation of complex B in the splicing process. Besides its role in the U5 splicing complex, *Snrnp200* plays an additional role as an activator of interferon beta (IFN-beta) in macrophages in the presence of viral RNA (Tremblay *et al.* 2016). SNRNP200 recognizes and binds with viral RNA and, together with its partner TBK1, activates the IFN-beta transcription factor, IRF3, which in turn promotes transcription of *Ifn-beta*. SNRNP200, IRF3, and *Ifn-beta* oscillate with highly similar waveforms to each other and to *clock*, and all peak during the inflammatory phase of the day as defined by (Collins *et al.* 2021) (Supplementary Fig. 3). This presents a possible mechanism for IFNb regulation, and perhaps IFN-beta-mediated regulation of inflammation, through the clock-control of spliceosome components. Beyond SNRNP200, ECHO identified 24/64 (37.5%) of proteins in the spliceosome complex oscillated with a circadian period, a larger proportion than was seen in the overall oscillating proteome (29%), with a significant discordance between the oscillating mRNA and oscillating proteins (Collins *et al.* 2021).

## Discussion

The recognition of novel oscillatory characteristics is essential for the study of circadian rhythms, and a new generation of analytical tools is needed to address the changing modeling requirements for this task (De los Santos *et al.* 2020; Patke *et al.* 2020). Key areas where current tools are lacking are in the ability to analyze different AC categories, the accessibility and usability of the software, the application of multiple hypothesis testing and joint modeling, and support for a wide range of model organisms (Smolen and Byrne 2009; Diz *et al.* 2011; Betini *et al.* 2017; De los Santos *et al.* 2021). We have created the PAICE suite as a tool to address all of these challenges and present here a comprehensive user tutorial of this unique oscillatory modeling software. The PAICE suite, which encompasses ECHO, ENCORE, and MOSAIC, is capable of modeling ACs and jointly modeling multiomics data sets; provides robust user-friendly interfaces and visualizations; offers several statistical corrections for multiple hypothesis testing and ontological analysis of well-studied organisms; and is readily available as an open-source project on GitHub (Fig. 1).

Here, we demonstrate the PAICE suite’s effectiveness by extending the findings from Collins *et al.* (2021) on the circadian regulation of murine macrophages (Collins *et al.* 2021). We revealed that, while accounting for the noise in proteomics data, there is still significant circadian posttranscriptional regulation occurring in mammalian macrophages (Fig. 2 and Supplementary Fig. 1) (Collins *et al.* 2021). This reinforces that the clock selectively targets cellular functions at specific times of day via posttranscriptional mechanisms, as has been previously suggested (Kojima *et al.* 2012; Caster *et al.* 2016; Wang *et al.* 2018; Zinn-Brooks and Roper 2021). We further demonstrated that specific ontologies were aligned with specific ACs (Fig. 3). In general, we predict that the extensive damping that we noted in the transcriptome, particularly the transcripts involved in immune ontologies, may be related to the depletion of nutrients from the media, driving the cells to enter a starvation response as the time course progresses (Fig. 3) (Collins *et al.* 2021). This finding aligns with previous evidence that the circadian clock participates in propagating the starvation response and the overall energy maintenance in immune cells (Pakos-Zebrucka *et al.* 2016; McAlpine and Swirski 2016; De los Santos *et al.* 2020; Collins *et al.* 2021).

A limitation to the ontological analysis program in the PAICE suite (i.e. ENCORE) is that currently it can only analyze proteins and protein-coding RNA, as its underlying repositories only support those analyses (De los Santos *et al.* 2019). This is a significant limitation as the circadian clock has been suggested to control ncRNA levels (Cui *et al.* 2015; Fan *et al.* 2017; Zhang *et al.* 2018; Hardeland 2020). While the correction of this issue is a future direction for the development of the PAICE suite, we coupled ECHO with noncoding ontological databases to investigate the regulation of ncRNAs in mammalian macrophages (Bao *et al.* 2019). We found that ncRNAs were more likely to be forced or harmonic, indicating a different circadian regulatory program on ncRNAs as compared to mRNAs. The circadian timing of ncRNAs is medically relevant, as lncRNAs tied to disease phenotypes were enriched among the circadian ncRNAs (Fig. 4). The role of lncRNAs in neurodegenerative disease is of particular note as our work has shown that macrophages play a role in the metabolism of amyloid beta; this suggests that lncRNAs may be a part of this metabolism (Clark *et al.* 2022).

Beyond the role in the health of an organism, the timing of ncRNAs may also be a primary mechanism by which the clock regulates posttranscriptional modification. lncRNAs have roles as *trans*-acting repressors of RNAPII, locus-specific silencing, and *cis-*acting chromosome inactivation, meaning that the timing of lncRNAs could regulate the timing of output (Kornienko *et al.* 2013). Beyond lncRNAs, analysis with the PAICE suite also revealed circadian regulation throughout the spliceosomal pathway, including the regulatory snRNA, mRNA, and proteins, which suggests ubiquitous clock control over regulation of the spliceosome. This likely indicates robustness in the clock’s ability to control splicing events, suggesting a further mechanism of circadian posttranscriptional regulation. However, further experiments directly targeting these genes and pathways will be required to validate these findings. While the functionality of the oscillating ncRNAs of interest is much debated, we provide the relative counts as well as raw TPM values for all ncRNAs in our dataset (Supplementary File 7). In total, the PAICE suite has allowed for a deeper understanding of the mechanisms by which the clock times mammalian macrophages and potentially other cell types.

## Data availability

All data are available on Mendeley data (https://data.mendeley.com/datasets/vrt3wdnf6y/2). Supplementary File 1 contains the ECHO analysis of all macrophage protein-coding RNAs. Supplementary File 2 contains the ECHO analysis of all macrophage proteins. Supplementary File 3 contains the list of proteins that required manual identification or deletion preceding analysis. Supplementary File 4 contains the ENCORE analysis of all macrophage protein-coding RNAs. Supplementary File 5 contains the ENCORE analysis of all macrophage proteins. Supplementary File 6 contains the MOSAIC analysis of the comparison between mRNA and protein. Supplementary File 7 contains raw TPM values of detected ncRNA. Supplementary File 8 contains the ECHO analysis of the ncRNA categories for pseudogenes and snoRNA. Supplementary File 9 contains the ECHO analysis of the ncRNA category for lncRNA. Supplementary File 10 contains a list of the circadian, disease-associated mouse lncRNAs analyzed using the LncRNADisease v2.0 database and their human orthologs. Supplementary File 11 contains the list of spliceosomal genes adapted from KEGG to identify the spliceosomal transcripts and proteins in the spliceosomal complexes. Supplementary File 12 contains the ECHO analysis of the ncRNA category for snRNA. Supplementary File 13 contains the NCBI annotation of all snRNAs. Supplementary Fig. 1 shows comparisons between ECHO and MOSAIC analyses of peak timing of circadian RNA and circadian protein. Supplementary Fig. 2 describes the AC categories and oscillations of the circadian lncRNA. Supplementary Fig. 3 describes the oscillation of the Snrnp200-IRF3B-Ifnb pathway.

Supplemental material is available at *G3* online.

## Supplementary Material

jkac176_Supplemental_Figure_1

jkac176_Supplemental_Figure_2

jkac176_Supplemental_Figure_3
