# Supplementary material for: The PAICE suite reveals circadian posttranscriptional timing of noncoding RNAs and spliceosome components in Mus musculus macrophages
Source: G3 (Bethesda). 2022 Jul 25;12(9):jkac176. doi: 10.1093/g3journal/jkac176 (PMC9434326; doi:10.1093/g3journal/jkac176)
Supplement: jkac176_Supplemental_Figure_1 [file jkac176_supplemental_figure_1.pdf]

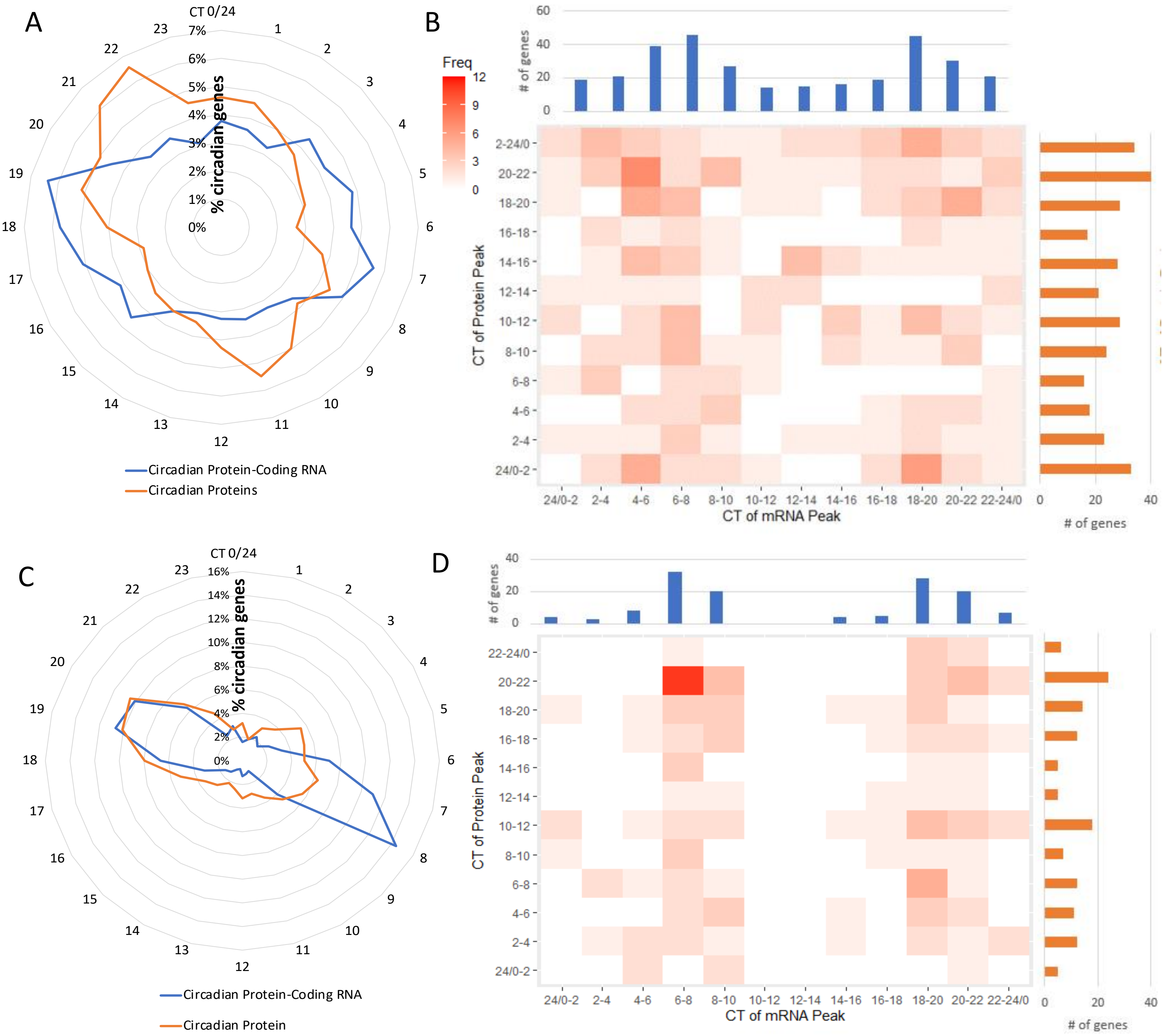

Supplemental Figure 1. MOSAIC analysis recapitulates the phase relationship of oscillating transcripts and proteins identified by ECHO. **(A)** The percentage of total oscillating transcripts (blue) or proteins (orange) peaking at a given time was plotted on a radial histogram by circadian time of peak binned in 1-h windows, as determined by ECHO analysis (shown as percentage of genes). **(B)** A heatmap comparing the difference in the peak phases of corresponding oscillating transcripts (blue) and proteins (orange) binned into 2-h intervals over circadian time for genes circadian at both the mRNA and protein level as determined by ECHO analysis. The histogram at the top of the graph shows the sum of the absolute number of mRNAs in each column, and the histogram on the right side of the graph shows the sum of the absolute number of proteins in each row. **(C)** The percentage of total oscillating transcripts (blue) or proteins (orange) peaking at a given time was plotted on a radial histogram by circadian time of peak binned in 1-h windows, as determined by MOSAIC analysis (shown as percentage of genes). **(D)** Transcripts (blue) and proteins (orange) binned into 2-h intervals over circadian time for genes circadian at both the mRNA and protein level as determined by MOSAIC analysis. The histogram at the top of the graph shows the sum of the absolute number of mRNAs in each column, and the histogram on the right side of the graph shows the sum of the absolute number of proteins in each row.
