# Supplementary material for: The PAICE suite reveals circadian posttranscriptional timing of noncoding RNAs and spliceosome components in Mus musculus macrophages
Source: G3 (Bethesda). 2022 Jul 25;12(9):jkac176. doi: 10.1093/g3journal/jkac176 (PMC9434326; doi:10.1093/g3journal/jkac176)
Supplement: jkac176_Supplemental_Figure_2 [file jkac176_supplemental_figure_2.pdf]

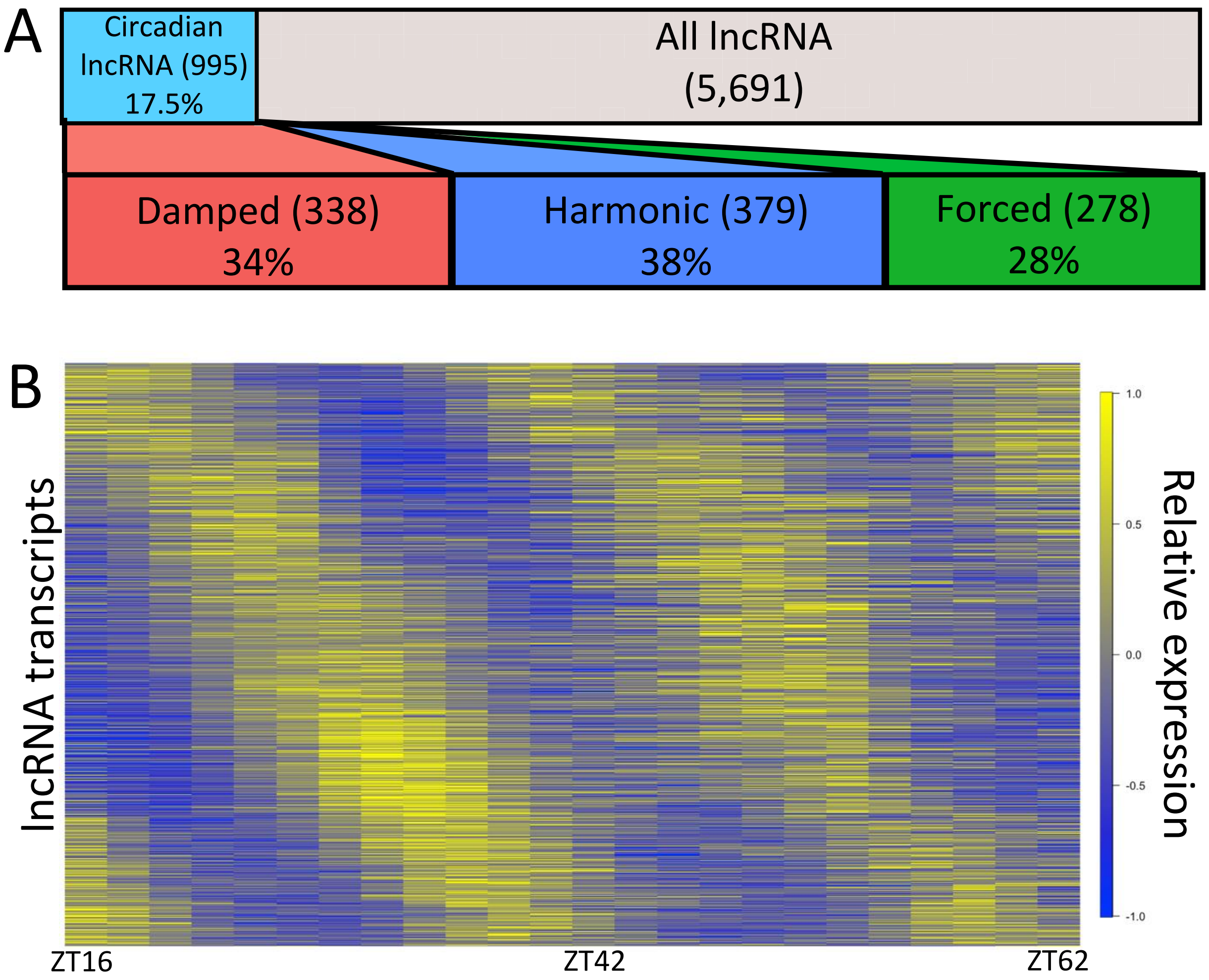

Supplemental Figure 2. LncRNAs display circadian rhythms. A) Displays the proportion of circadian lncRNAs to all detected lncRNAs and breakdown of AC categories of circadian lncRNAs (absolute numbers in parentheses). B) Displays a heatmap derived from ECHO displaying identified circadian rhythms in 995 lncRNAs over 48hrs of circadian time (period restriction =20-28hrs, BH-adj p-value = <0.05; ZT = hours post serum shock). Data adapted from Collins et. al, 2021.
