# Supplementary material for: The PAICE suite reveals circadian posttranscriptional timing of noncoding RNAs and spliceosome components in Mus musculus macrophages
Source: G3 (Bethesda). 2022 Jul 25;12(9):jkac176. doi: 10.1093/g3journal/jkac176 (PMC9434326; doi:10.1093/g3journal/jkac176)
Supplement: jkac176_Supplemental_Figure_3 [file jkac176_supplemental_figure_3.pdf]

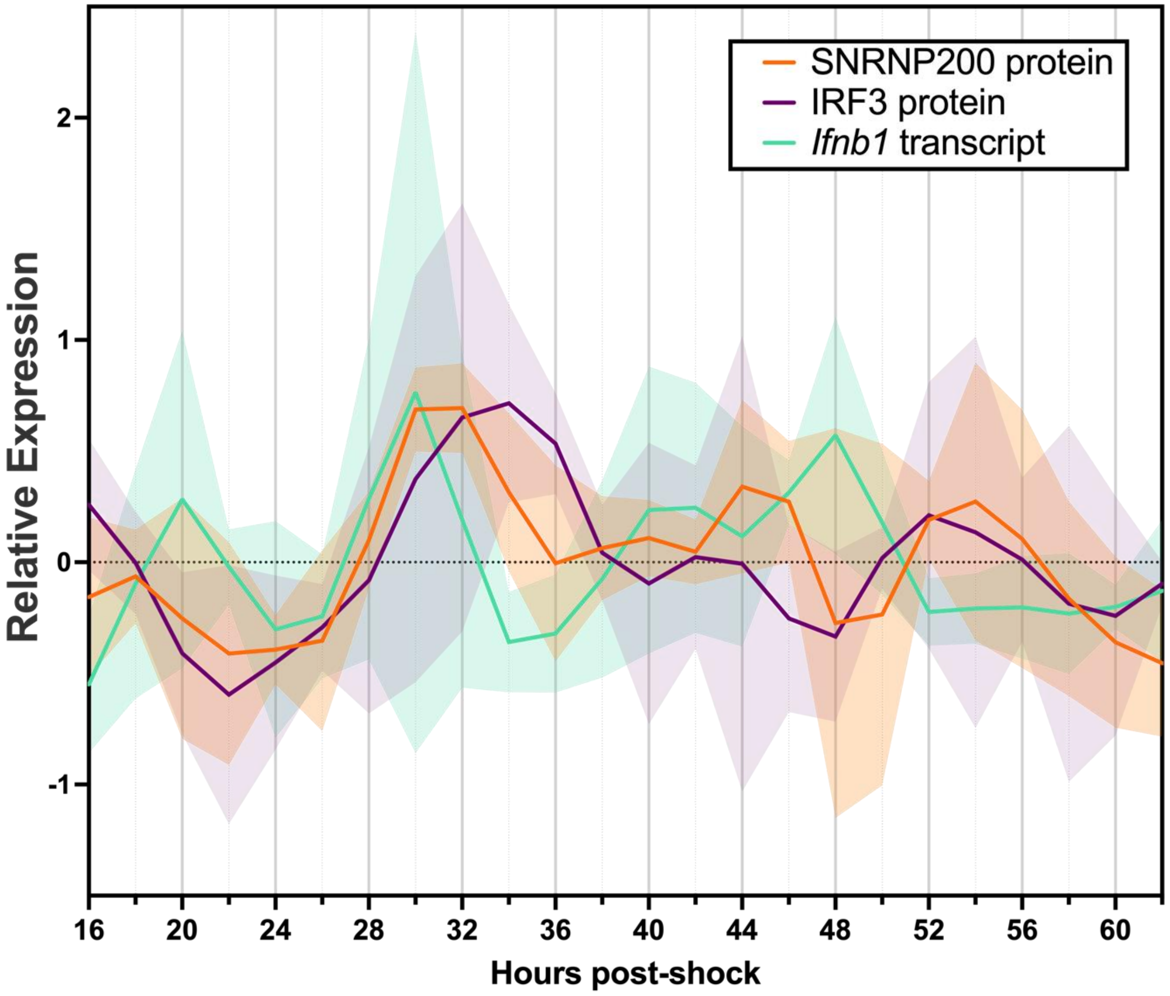

Supplemental Figure 3. Circadian components of Ifnb1 activation pathway oscillate in-phase. ECHO-modeled curve and shaded standard deviation of components for two proteins implicated in Ifnb expression, and Ifnb transcript. X-axis refers to hours following serum shock as referenced in Collins et. al, 2021. ECHO values for each gene: Snrnp200, period =20.6hr, BH-adj p-value 1.99e-03; IRF3 period = 21.4hr, BH-Adj p-value = 3.18e-05; Ifnb1 period = 20.5hr, BH-adj p-value = 1.33e-03. Data adapted from Collins et. al, 2021.
